# VCP Is an Integral Component of a Novel Feedback Mechanism that Controls Intracellular Localization of Catalase and H_2_O_2_ Levels

**DOI:** 10.1371/journal.pone.0056012

**Published:** 2013-02-14

**Authors:** Katsuhiro Murakami, Yuzuru Ichinohe, Masaaki Koike, Norio Sasaoka, Shun-ichiro Iemura, Tohru Natsume, Akira Kakizuka

**Affiliations:** 1 Laboratory of Functional Biology, Kyoto University Graduate School of Biostudies, Sakyo-ku, Kyoto, Japan; 2 National Institutes of Advanced Industrial Science and Technology, Biological Information Research Center (JBIRC), Kohtoh-ku, Tokyo, Japan; Institute for Virus Research, Laboratory of Infection and Prevention, Japan

## Abstract

Catalase is a key antioxidant enzyme that catalyzes the decomposition of hydrogen peroxide (H_2_O_2_) to water and oxygen, and it appears to shuttle between the cytoplasm and peroxisome via unknown mechanisms. Valosin-containing protein (VCP) belongs to the AAA class of ATPases and is involved in diverse cellular functions, e.g. cell cycle and protein degradation, etc. Here we show that VCP and PEX19, a protein essential for peroxisome biogenesis, interact with each other. Knockdown of either VCP or PEX19 resulted in a predominantly cytoplasmic redistribution of catalase, and loss of VCP ATPase activity also increased its cytoplasmic redistribution. Moreover, VCP knockdown decreased intracellular ROS levels in normal and H_2_O_2_-treated cells, and an oxidation-resistant VCP impaired the ROS-induced cytoplasmic redistribution of catalase. These observations reveal a novel feedback mechanism, in which VCP can sense H_2_O_2_ levels, and regulates them by controlling the localization of catalase.

## Introduction

Reactive oxygen species (ROS), e.g. superoxide radicals, hydrogen peroxide, etc., are natural byproducts of the aerobic metabolism of foods, and they have been shown to play important roles in several physiological functions, e.g. transcriptional regulation, mitogen signaling, integrin signaling, Wnt signaling, etc. (see refs in [1]–[3]). On the other hand, ROS are also produced by UV and X-ray exposure or inflamation, and excess ROS can damage cellular functions by oxidizing proteins, lipids, and DNA, leading to cell aging as well as cancer (see refs in [4]). Among ROS, the superoxide radical is enzymatically converted by superoxide dismutases (SODs) to hydrogen peroxide (H_2_O_2_), which, in turn, is converted by catalase or peroxidases to H_2_O and O_2_. Mammalian cells typically possess three SODs, several peroxidases, and one catalase. Among these ROS-scavenging enzymes, only catalase resides in peroxisomes. In certain conditions, such as aging, catalase also resides in the cytoplasm [5], which is believed to be due to its weak peroxisome-targeting signal (PTS). Two types of PTS, PTS1 and PTS2, are known [6]. Typically, PTS1 consists of three sequential amino acids, SKL, and it is present in peroxisome-localized proteins such as peroxisomal Acyl-CoA thioesterase, PTE1. Catalase has a unique PTS1, consisting of four sequential amino acids, KANL. Both PTS1s are recognized by PEX5 (Peroxisome biogenesis factor 5); however, PEX5 binds to SKL more strongly than to KANL [7], and thus it is believed that PEX5 can transfer SKL-containing proteins more effectively than catalase to peroxisomes. In aged cells, cellular levels of ROS increase, and it is thought that such ROS may weaken PEX5 functions, with transport of catalase to peroxisomes being preferentially compromised, as opposed to transport of SKL-possessing proteins [8]. However, no clear evidence has been provided supporting this speculation.

VCP belongs to the AAA class of ATPases and has been shown to function in many cellular events, including ERAD (endoplasmic reticulum-associated degradation), cell cycle control, membrane fusion, maintenance of Golgi apparatus, protein aggregate formation and clearance, etc. (see refs in [9]). VCP has also been shown to play important roles in several human neurodegenerative disorders [10]–[12]. We have shown that VCP is modified post-translationally at 60 amino acids, at least, including 18 serines, 14 threonines, 6 tyrosines, and 22 lysines [13]. To investigate the role of post-transcriptional modifications of VCP, we created several modification-mimic forms of VCP, and characterized them [12]–[14]. These analyses have revealed novel VCP functions and have led us to speculate that VCP may have unidentified functions. In this study, we report a novel VCP function in regulation of intracellular H_2_O_2_ levels via the control of catalase localization.

## Materials and Methods

### Antibodies

The following antibodies were purchased: anti-actin (Chemicon), anti-catalase (Calbiochem), anti-PTE1 (ACOT8) (Santa Cruz), anti-FLAG M2 (Sigma), anti-PMP70 (Zymed), anti-HA (Santa Cruz), and anti-PEX19 (BD PharMingen). The affinity-purified rabbit polyclonal anti-VCP antibody was described previously [15].

### Plasmids and siRNAs

The cDNAs for PEX5, PEX19, catalase, and PTS2 signal sequences of ACAA1 (acetyl-CoA acyltransferase 1) were amplified by RT–PCR from total RNA isolated from HeLa cells, and their sequences were confirmed. The VCP cDNAs (wtVCP, VCP[K251A], VCP[K524A] [16]) or PEX5 cDNA was subcloned into pmCherry vector (Clontech). The PEX19 cDNA was subcloned into pCMV-HA vector (Clontech).

The targeting sequences of siRNAs for VCP and PEX5 mRNAs were as follows:

VCP(nc), 5′-CGGGAGAGGCGCGCGCCAT-3′;

VCP(286), 5′-GGTTAATTGTTGATGAAGCCATCAA-3′;

PEX5(192), 5′-CAAGCCTTTGGGAGTAGCTTCTGAA-3′;

PEX5(955), 5′-GACCTTACGTCAGCTACCTATGATA-3′.

Control, 5′-CGGACGCGTCAGGAGCCGGTT-3′.

The siRNAs for PEX19 were purchased from Invitrogen (Stealth Select RNAi, HSS108913 and HSS108914, respectively).

### Cell Culture and Cell Lines

HeLa cells and HEK293A cells were grown at 37°C in Dulbecco’s modified Eagle’s medium supplemented with 10% fetal bovine serum. HeLa cell lines stably expressing organelle-targeted GFPs were created by transfection of organelle-targeted GFP expression vectors, and selected in the presence of 2.5 µg/ml of puromycin (Invivogen). The HEK293A cell line stably expressing GFP-catalase, was also created by similar methods.

### Transfection and Immunostaining

Plasmid transfection was carried out using Lipofectamine plus (Invitrogen), and siRNA transfection was carried out using Oligofectamine (Invitrogen) according to the manufacturer’s protocol. In co-transfection experiments, cells were transfected with siRNA and plasmid using Lipofectamine 2000 (Invitrogen).

Cells were fixed with 4% formaldehyde for 10 min at room temperature. Fixed cells were permeabilized with 0.5% Triton X-100 in PBS for 10 min at room temperature and blocked with blocking buffer (0.1% bovine serum albumin and 0.1% skim milk in PBS) for 1 h. Cells were then incubated 1 h at room temperature with primary antibodies. Subsequently, cells were treated with Alexa Fluor 488-conjugated secondary antibodies (Invitrogen). To detect PMP70, fixed cells were permeabilized with 25 µg/ml digitonin in PBS for 5 min at room temperature, and cells were processed for immunostaining as describe above.

### Subcellular Fractionation and Immunoprecipitation

Cells were fractionated into cytosol, membrane/organelle, and nucleus, using a Subcellular Proteome extraction kit (Calbiochem), according to the manufacturer's protocol. Immunoprecipitation assays were performed as described previously [16]. Briefly, samples were lysed on ice and debris was removed by centrifugation for 30 min at 15,000×g at 4°C. The supernatant was mixed with an anti-HA or anti-FLAG antibody and rotated at 4°C overnight after addition of protein G-Sepharose beads (Amersham Biosciences). After washing of beads, bound proteins were analyzed by Western blot.

### Intracellular ROS Detection

Cells were washed twice with HBSS and incubated with 5 µM CM-H_2_DCFDA, a ROS-detection reagent (Invitrogen), in HBSS at 37°C for 30 min. Subsequently, cells were washed twice with HBSS and incubated with growth medium at 37°C for 30 min with or without H_2_O_2_. Then cells were analyzed by FACScan flow cytometer (BD Biosciences) or LSM510 confocal microscopy (Carl Zeiss).

### Statistical Analysis

Each experiment was conducted at least three times with consistent results. The gel or blot representative of each experiment is presented in this study. The statistical significance was analyzed using Student’s *t* test.

## Results

### Involvement of VCP in Intracellular Localization of Catalase

**Figure 1:**
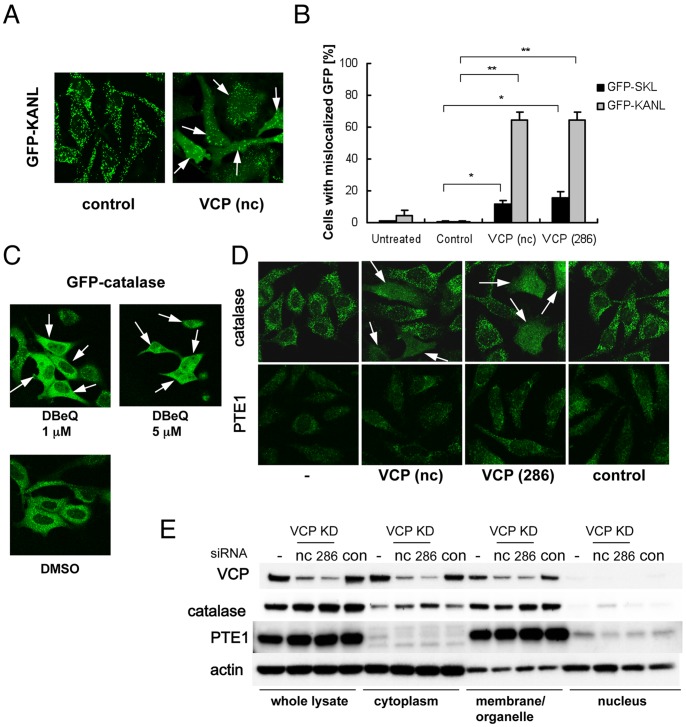
VCP siRNAs induce cytoplasmic localization of catalase. (A) Cytochemical analysis of intracellular localization of GFP-KANL. HeLa cells continuously expressing GFP-KANL were treated with control siRNA (control) or VCP siRNAs (nc and 286). Seventy-two hours later, GFP images were analyzed by confocal microscopy. Arrows indicate cells with cytoplasmic localization of GFP-KANL. (B) Quantification of cytochemical analysis on GFP-KANL in (A) and on GFP-SKL. More than 200 cells were examined in each sample, and the fraction (%) of cells with diffuse GFP signals in the cytoplasm were scored. **p<0.01, *p<0.05. (C) Fluorescence microscopy analysis of intracellular localization of GFP-catalase. HEK293A cells continuously expressing GFP-catalase were treated with 1 µM or 5 µM DBeQ, a VCP inhibitor [17], or DMSO for 24 hours, and then GFP signals were detected. Arrows indicate cells with cytoplasmic localization of GFP-catalase. (D) Immunocytochemical analysis of intracellular localization of catalase and PTE1. HeLa cells were treated without (−) or with control siRNA (control), or VCP siRNAs (nc and 286). Seventy-two hours later, catalase and PTE1 were detected with anti-catalase and anti-PTE1 antibodies, respectively. Arrows indicate cells with cytoplasmic localization of catalase. (E) Western blot analyses of protein levels of VCP, catalase, and PTE1 in different cell compartments. HeLa cells were treated without (−) or with control siRNA (control) or VCP siRNAs (nc and 286). Seventy-two hours later, cells were fractionated as described in Methods. Fractionated samples equivalent to 7.5 µg total protein of whole cell lysates were separated by SDS-PAGE and analyzed by western blotting using specific antibodies. Actin served as a loading control.

In order to visualize organelle in live cells, we generated several HeLa cell sublines in which GFP was expressed as a fusion protein with a peroxisomal (PTS1 or PTS2)-, nuclear (NLS)-, ER (KDEL)-, or mitochondrial (mito)-targeting signal. Among these, we observed clear mislocalization of GFP-PTS1 (namely, GFP-SKL and GFP-KANL) into the cytoplasm when the cells were treated with VCP siRNAs but not a control siRNA. VCP siRNAs perturbed GFP-KANL localization much more severely than GFP-SKL localization (**Fig. 1A and B**). By contrast, VCP siRNAs did not induce clear mislocalization of PTS2-GFP, mito-GFP, GFP-ER, or GFP-NLS (Fig. S1). We observed similar mislocalization of GFP-KANL by expressing ATPase-negative or dominant-negative VCP mutants, e.g. VCP[K251A] and VCP[K524A] [16] (Fig. S2). In addition, treating cells with DBeQ, a VCP inhibitor [17], also induced cytoplasmic localization of GFP-catalase (**Fig. 1C**). These results suggest that the ATPase activity of VCP is necessary for proper localization of catalase.

We then examined the effects of VCP knockdown on intracellular localization of endogenous PTE1 or catalase. In more than 50% of cells treated with VCP siRNAs, endogenous catalase was diffusely observed in the cytoplasm. In contrast, PTE1 localization was not apparently affected by VCP knockdown (**Fig. 1D**). These results were confirmed by cell fractionation experiments. VCP siRNA treatments increased the amounts of catalase but not PTE1 in the cytoplasmic fraction (**Fig. 1E**). Mislocalization of catalase as well as GFP-KANL decreased in cells treated with VCP siRNA together with cycloheximide (Fig. S3), supporting the idea that newly synthesized catalase is transported into peroxisomes with the help of VCP.

### Interaction between VCP and PEX19

**Figure 2:**
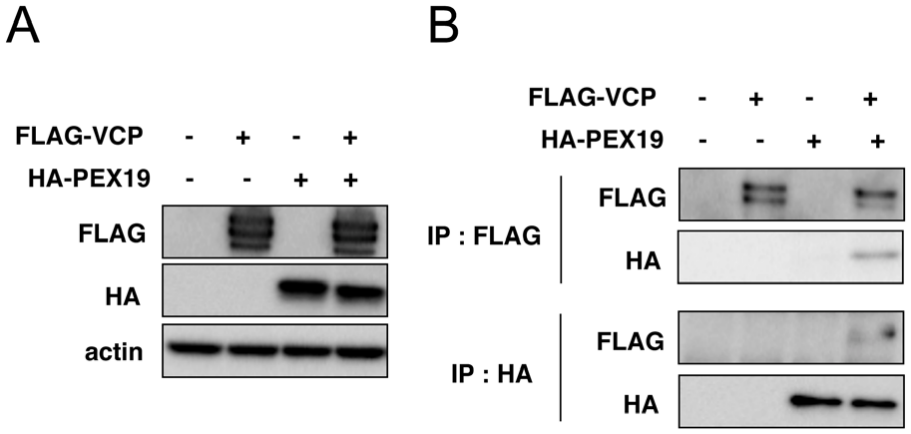
Immunoprecipitation assays to detect physical interactions between VCP and PEX19. (A) HEK293A cells were transfected with expression vectors for FLAG-VCP and HA-PEX19. Twenty-four hours later, cells were harvested and the cell lysates were analyzed by western blots with antibodies indicated in the panels. Actin served as a loading control. (B) The immunoprecipitation was performed on the cell lysates in (A) with an anti-FLAG or anti-HA antibody. The precipitates were analyzed by western blots with antibodies indicated in the panels. See details in **Materials and methods.**

In order to obtain insights for molecular mechanisms underlying VCP-mediated regulation of catalase localization, we searched for VCP-interacting proteins using an immunoprecipitation method followed by a very sensitive MS/MS analysis [18], and identified PEX19 as a potential VCP-interacting protein. Indeed, we could observe a physical association between VCP and PEX19 via immunoprecipitation and western blotting (**Fig. 2**). This interaction appeared very weak, suggesting the possibility that yet-unknown VCP modification may enhance this interaction. This possibility remained to be clarified.

### Involvement of PEX19 in Intracellular Localization of Catalase

**Figure 3:**
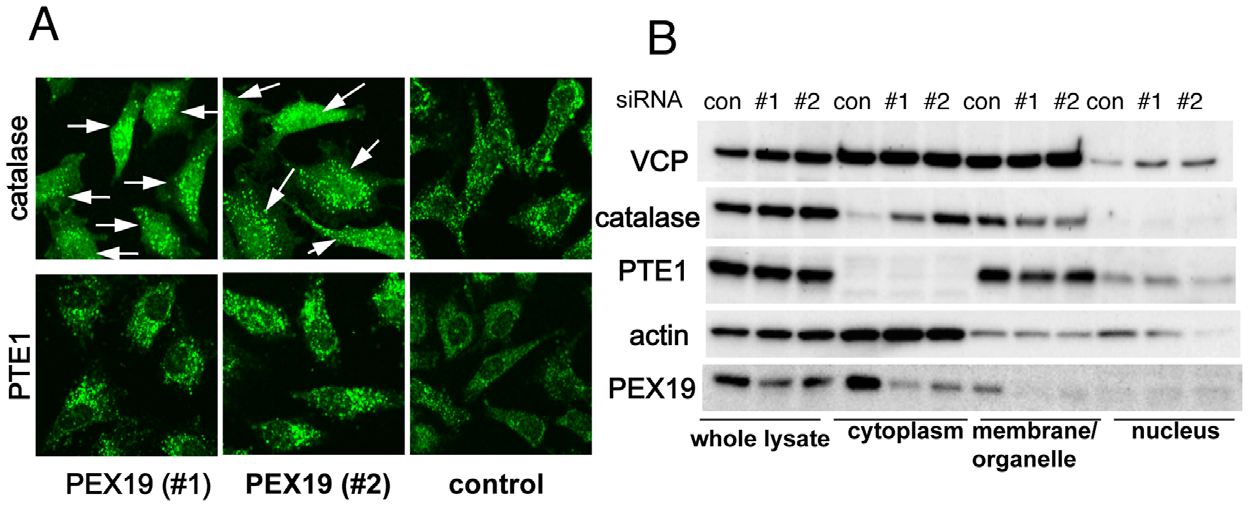
PEX19 siRNAs also induce cytoplasmic localization of catalase. (A) Immunocytochemical analysis of intracellular localization of catalase and PTE1. HeLa cells were treated with control siRNA (control) or PEX19 siRNAs (#1; HSS108913 and #2; HSS108914). Seventy-two hours later, catalase and PTE1 were detected as in (Fig. 1D). Arrows indicate cells with cytoplasmic localization of catalase. (B) Western blot analyses of protein levels of VCP, catalase, PTE1, and PEX19 in different cell compartments. HeLa cells were treated with control siRNA (control) or PEX19 siRNAs (#1; HSS108913 and #2; HSS108914). Seventy-two hours later, cells were analyzed as in (Fig. 1E).

Given that VCP could potentially make a complex with PEX19 and that VCP knockdown apparently affected the transport of catalase into peroxisomes, PEX19 knockdown could also affect the intracellular localization of catalase. Indeed, PEX19 knockdowns produced virtually identical distributions of intracellular catalase as were observed in VCP knockdowns (**Fig. 3A and B**). Moreover, in PEX19 knockdown cells, PTE1 localization was not apparently affected (**Fig. 3A and B**). PEX19 is reportedly involved in the transport of membrane proteins, such as PMP70 (peroxisome membrane protein 70), to peroxisomes. However, we could not detect any clear mislocalization of endogenous PMP70 in VCP-depleted HeLa cells (Fig. S4).

Consistent with previous reports, PEX5 knockdown induced mislocalization of both catalase and PTE1 (Fig. S5). It is notable that over-expression of PEX5 and VCP could not rectify the mislocalization of GFP-KANL in VCP and PEX5 knockdown cells, respectively (Fig. S6). These results indicate that VCP/PEX19 complexes are required for PEX5 to transport catalase, but not other typical PTS1- or PTS2-possessing proteins, to peroxisomes.

### VCP Activity, Catalase Localization, and ROS Levels

**Figure 4:**
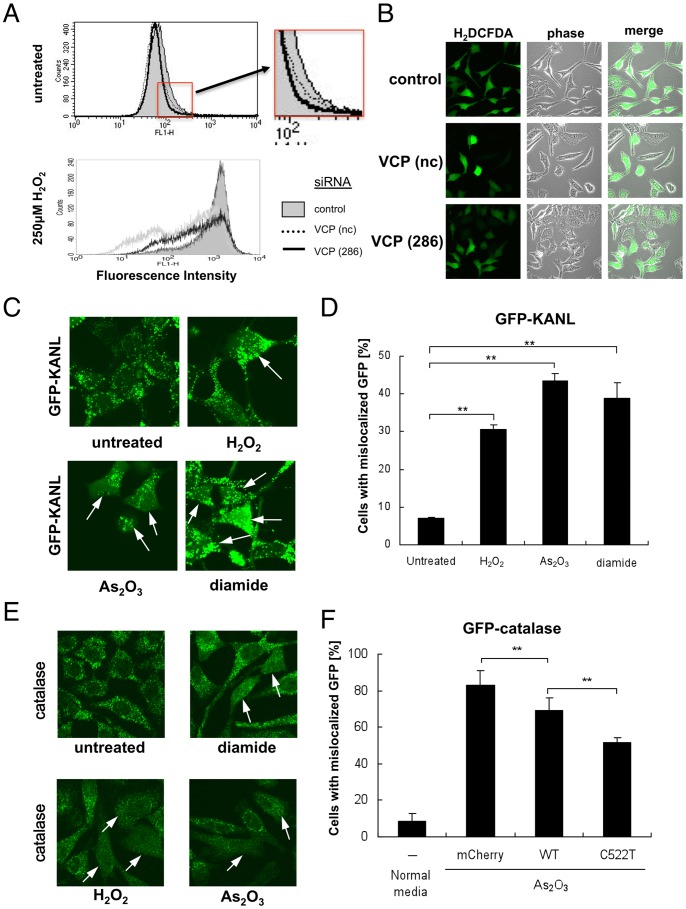
ROS levels and intracellular localization of GFP-KANL and catalase in cells with VCP depletion or overexpression. (A) ROS levels in cells with VCP depletion. HeLa cells were treated with control (control) or VCP (nc and 286) siRNAs. Seventy-two hours later, 5 µM H_2_DCFDA was added to the media for 30 min. Then, cells were treated with or without 250 µM H_2_O_2_ at 37°C for additional 30 min, and analyzed by FACS (see details in **Materials and methods**). (B) Cytochemical analysis of HeLa cells treated with 250 µM H_2_O_2_. H_2_DCFDA fluorescence was analyzed by confocal microscopy as in (A). (C) Cytochemical analysis of intracellular localization of GFP-KANL after treatment of ROS-producing agents. HEK293A cells continuously expressing GFP-KANL were treated with 250 µM H_2_O_2_, 20 nM As_2_O_3_, or 250 µM diamide. Twenty-four hours later, GFP images were analyzed by confocal microscopy. Arrows indicate cells with cytoplasmic localization of GFP-KANL. (D) Quantification of cytochemical analysis in (C). More than 200 cells were examined in each sample, and the fraction (%) of cells with diffuse GFP signals in the cytoplasm were scored. **p<0.01. (E) Immunocytochemical analysis of intracellular localization of catalase after treatment with ROS-producing agents. HeLa cells were treated with 500 µM H_2_O_2_, 20 nM As_2_O_3_, or 250 µM diamide. Twenty-four hours later, catalase was detected with an anti-catalase antibody. Arrows indicate cells with cytoplasmic localization of catalase. (F) Overexpression of VCP[C522T] weakened cytoplasmic localization of catalase by ROS more significantly than that of wild-type VCP. HEK293A cells continuously expressing GFP-catalase were transfected with VCP[C522T]-mCherry (C522T) or wild-type VCP-mCherry (wtVCP), and treated with 20 nM As_2_O_3_ for 24 hours. More than 200 mCherry-positive cells were examined in each sample, and the fraction (%) of cells with diffuse GFP signals in the cytoplasm were scored. **p<0.01.

We next examined the possibility that VCP-depleted cells have a greater capacity to scavenge H_2_O_2_ as compared with non-treated cells, due to the presence of catalase in the cytoplasm. This was indeed the case. Basal ROS levels were reduced in cells treated with VCP siRNAs compared to those treated with control siRNAs (**Fig. 4A**). Reduction of ROS levels was more pronounced when cells were treated with H_2_O_2_ (**Fig. 4A and B**). We have previously shown that the ATPase activity of VCP was inactivated by oxidation of Cys522 by ROS, such as H_2_O_2_ [14]. The observation that ATPase activity of VCP is necessary for proper catalase localization to peroxisomes raised the possibility that ROS treatments would also induce redistribution of catalase. We next examined this possibility, and confirmed that all tested ROS-inducing agents (such as H_2_O_2_, As_2_O_3_, and diamide) induced cytoplasmic localization of GFP-KANL as well as catalase (**Fig. 4C–E**). We then examined whether VCP[C522T], a VCP mutant with the ROS-sensitive cysteine to threonine substitution [14], had protective effects on redistribution of catalase in cells treated with ROS. Indeed, overexpression of VCP[C522T] significantly inhibited cytoplasmic localization of catalase in cells treated with As_2_O_3_, compared to overexpressed wild-type VCP (**Fig. 4F** **)**.

## Discussion

The results presented in this study, taken together, point to the existence of a novel feedback mechanism: when H_2_O_2_ levels increase, VCP ATPase is inactivated by Cys522 oxidation, which in turn keeps catalase in the cytoplasm, leading to reduced H_2_O_2_ levels. After H_2_O_2_ levels are reduced, glutathione as well as thioredoxine levels would recover, which would then restore VCP ATPase activity, leading to catalase transport into peroxisomes. This VCP-mediated system has the great merit of specifically changing the localization of catalase without affecting the localization of other peroxisome proteins.

In *S. cerevisiae,* Cys522 is not conserved in Cdc48p, a VCP homologue [14]. In what appears to be an evolutionary alternative design, *S. cerevisiae* possesses two catalases, one of which resides in peroxisomes and the other in the cytoplasm [19], [20]. *C. elegans* also possesses two catalases, one in peroxisomes and the other in the cytoplasm [21]. These lines of evidence strongly indicate that for living organisms catalase is needed in both peroxisomes and the cytoplasm. In mammals, a certain level of ROS, namely H_2_O_2_, is utilized in several physiological conditions, and, therefore the continuous presence of catalase in the cytoplasm might not be favorable. On the other hand, when mammalian cells meet conditions with a large amount of H_2_O_2_ in the cytoplasm, catalase would more effectively degrade and reduce H_2_O_2_ by accumulating in the cytoplasm. Thus, mammals have developed an integrated system to utilize one catalase rather than to have two differently localized catalases.

## Supporting Information

Figure S1 **Fluorescence microscopy analysis of intracellular localization of PTS2-GFP, mito-GFP, GFP-ER, and GFP-NLS.** (A) Schematic drawings of GFP-fused proteins. (B) HeLa cells were treated with control siRNA (control) or VCP siRNAs (nc and 286). Seventy-two hours later, GFP signals were detected.
(TIFF)

Figure S2 **Fluorescence microscopy analysis of intracellular localization of GFP-KANL in the presence of ATPase activity-defective mutant VCPs.** (A) HEK293A cells continuously expressing GFP-KANL were transfected with an expression vectors for mCherry or VCP (wtVCP, VCP[K251A] [15], or VCP[K524A] [15])-mCherry. Forty-eight hours later, GFP signals were detected. (B) Quantification of fluorescence microscopy of GFP-KANL in (A). More than 200 mCherry-positive cells were examined in each sample, and the fraction (%) of cells with diffuse GFP signals in the cytoplasm were scored. **p<0.01, *p<0.05.
(TIFF)

Figure S3 **Immunocytochemical and fluorescence microscopy analyses of intracellular localization of catalase and GFP-KANL.**
(A) HeLa cells were treated with control siRNA (control) or VCP siRNA (286) for 72 hours, and treated with or without cyclohexamide (CHX) (5 µg/ml) for additional 24 hours. Then catalase was detected with anti-catalase antibody. (B) HeLa cells continuously expressing GFP-KANL were treated with VCP siRNA (286). Cells were treated with or without 5 µg/ml of CHX from 48 (24 h) or 24 (48 h) to 72 hours after siRNA treatment. Then, GFP signals were detected.
(TIFF)

Figure S4 **Immunocytochemical analysis of intracellular localization of PMP70.** HeLa cells were treated without (−) or with control siRNA (control), or VCP siRNAs (nc and 286). Seventy-two hours later, PMP70 was detected with an anti-PMP70 antibody. Note that VCP protein levels decreased by VCP siRNA treatments, as shown in Fig. 1E
(TIFF)

Figure S5 **Immunocytochemical analysis of intracellular localization of catalase and PTE1.** HeLa cells were treated with control siRNA (control) or PEX5 siRNAs (192 and 955). Seventy-two hours later, catalase and PTE1 were detected with anti-catalase and anti-PTE1 antibodies, respectively.
(TIFF)

Figure S6 **Fluorescence microscopy analysis of intracellular localization of GFP-KANL.** (A) HeLa cells continuously expressing GFP-KANL were treated with control siRNA (control), VCP siRNA (nc), or PEX5 siRNA (192) for 48 hours, and then transfected with an expression vector for mCherry, VCP-mCherry, or mCherry-PEX5. Twenty-four hours later, GFP signals (green) and mCherry signals (red) were examined. (B) Quantification of fluorescence microscopy of GFP-KANL in (A). More than 120 mCherry-positive cells were examined in each sample, and the fraction (%) of cells with diffuse GFP signals in the cytoplasm were scored. n.s., not significant.
(TIFF)
