# Supplementary figures and images for: VCP Is an Integral Component of a Novel Feedback Mechanism that Controls Intracellular Localization of Catalase and H2O2 Levels
Source: PLoS One. 2013 Feb 14;8(2):e56012. doi: 10.1371/journal.pone.0056012 (PMC3573100; doi:10.1371/journal.pone.0056012)

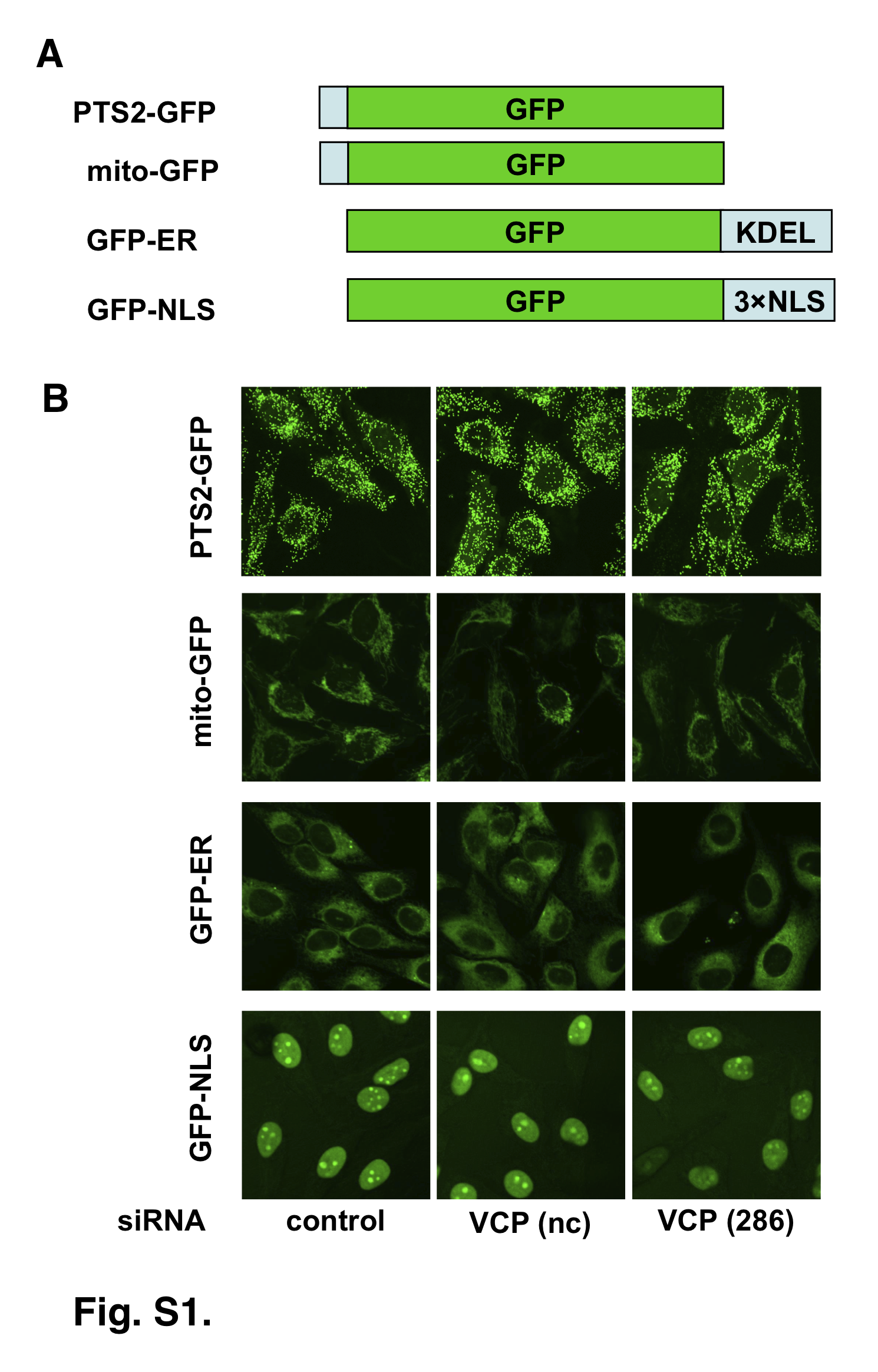

Supplement: Figure S1 — Fluorescence microscopy analysis of intracellular localization of PTS2-GFP, mito-GFP, GFP-ER, and GFP-NLS. (A) Schematic drawings of GFP-fused proteins. (B) HeLa cells were treated with control siRNA (control) or VCP siRNAs (nc and 286). Seventy-two hours later, GFP signals were detected. (TIFF) [file pone.0056012.s001.tiff]

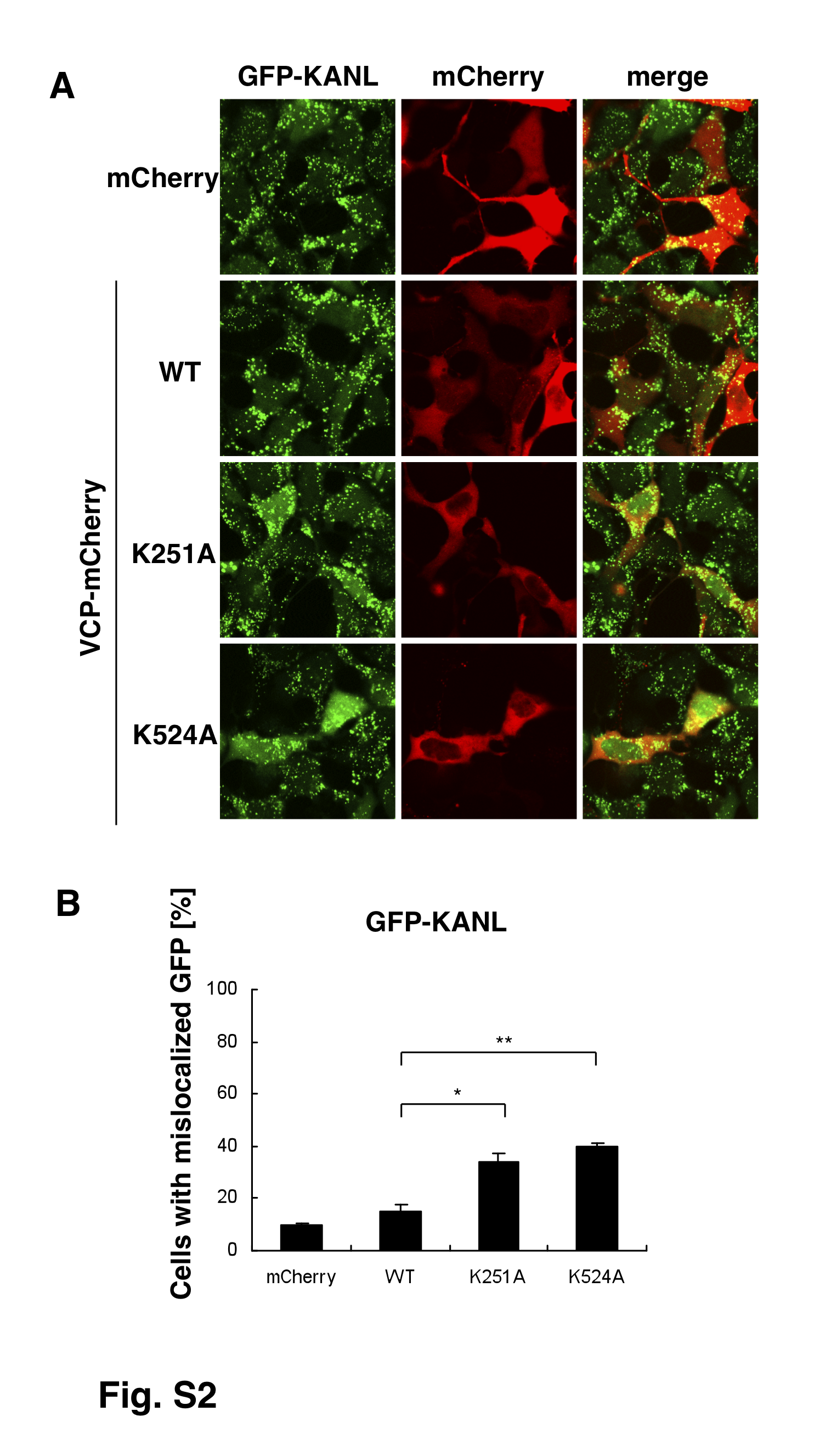

Supplement: Figure S2 — Fluorescence microscopy analysis of intracellular localization of GFP-KANL in the presence of ATPase activity-defective mutant VCPs. (A) HEK293A cells continuously expressing GFP-KANL were transfected with an expression vectors for mCherry or VCP (wtVCP, VCP[K251A] [15], or VCP[K524A] [15])-mCherry. Forty-eight hours later, GFP signals were detected. (B) Quantification of fluorescence microscopy of GFP-KANL in (A). More than 200 mCherry-positive cells were examined in each sample, and the fraction (%) of cells with diffuse GFP signals in the cytoplasm were scored. **p<0.01, *p<0.05. (TIFF) [file pone.0056012.s002.tiff]

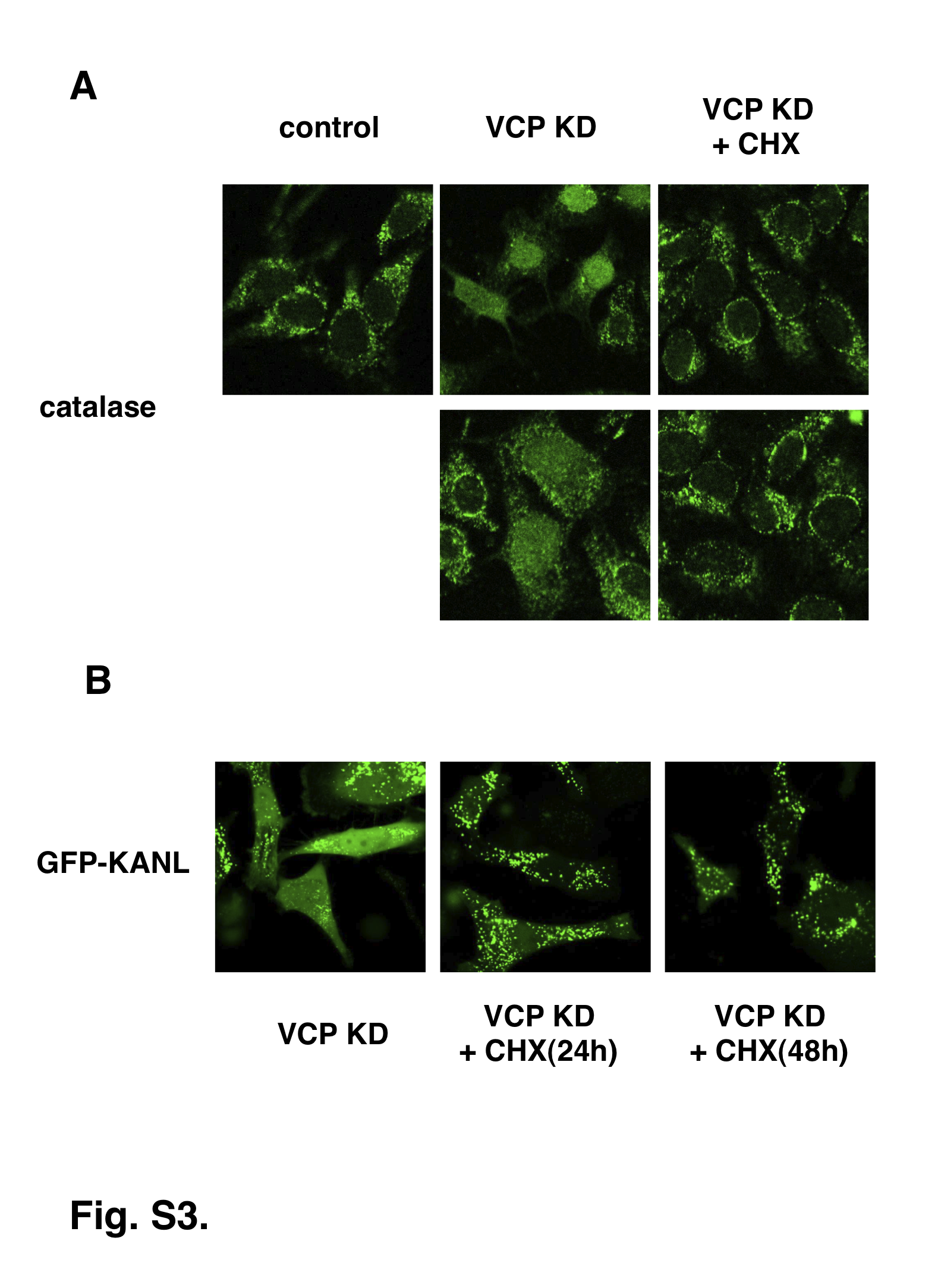

Supplement: Figure S3 — Immunocytochemical and fluorescence microscopy analyses of intracellular localization of catalase and GFP-KANL. (A) HeLa cells were treated with control siRNA (control) or VCP siRNA (286) for 72 hours, and treated with or without cyclohexamide (CHX) (5 µg/ml) for additional 24 hours. Then catalase was detected with anti-catalase antibody. (B) HeLa cells continuously expressing GFP-KANL were treated with VCP siRNA (286). Cells were treated with or without 5 µg/ml of CHX from 48 (24 h) or 24 (48 h) to 72 hours after siRNA treatment. Then, GFP signals were detected. (TIFF) [file pone.0056012.s003.tiff]

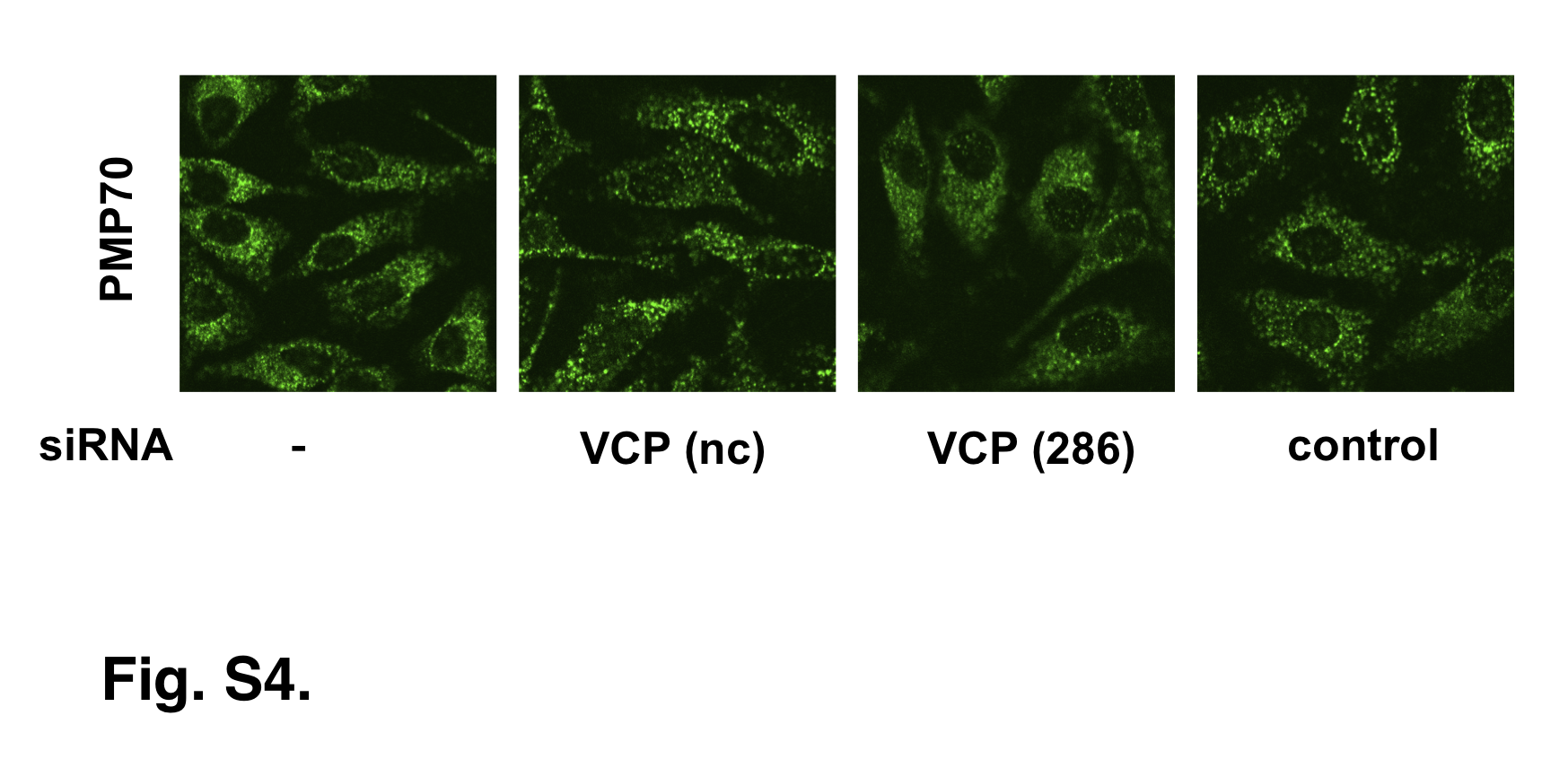

Supplement: Figure S4 — Immunocytochemical analysis of intracellular localization of PMP70. HeLa cells were treated without (−) or with control siRNA (control), or VCP siRNAs (nc and 286). Seventy-two hours later, PMP70 was detected with an anti-PMP70 antibody. Note that VCP protein levels decreased by VCP siRNA treatments, as shown in Fig. 1E (TIFF) [file pone.0056012.s004.tiff]

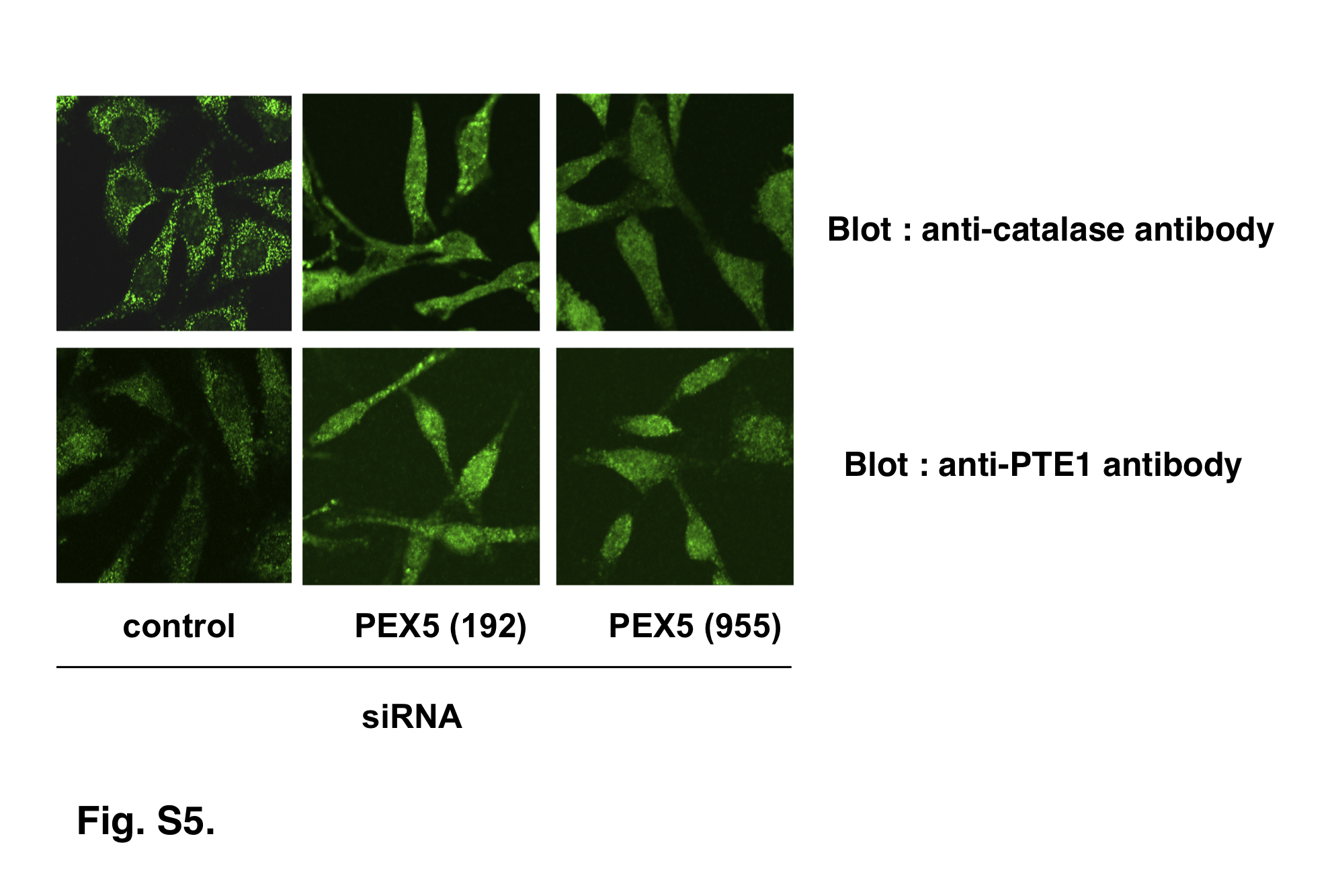

Supplement: Figure S5 — Immunocytochemical analysis of intracellular localization of catalase and PTE1. HeLa cells were treated with control siRNA (control) or PEX5 siRNAs (192 and 955). Seventy-two hours later, catalase and PTE1 were detected with anti-catalase and anti-PTE1 antibodies, respectively. (TIFF) [file pone.0056012.s005.tiff]

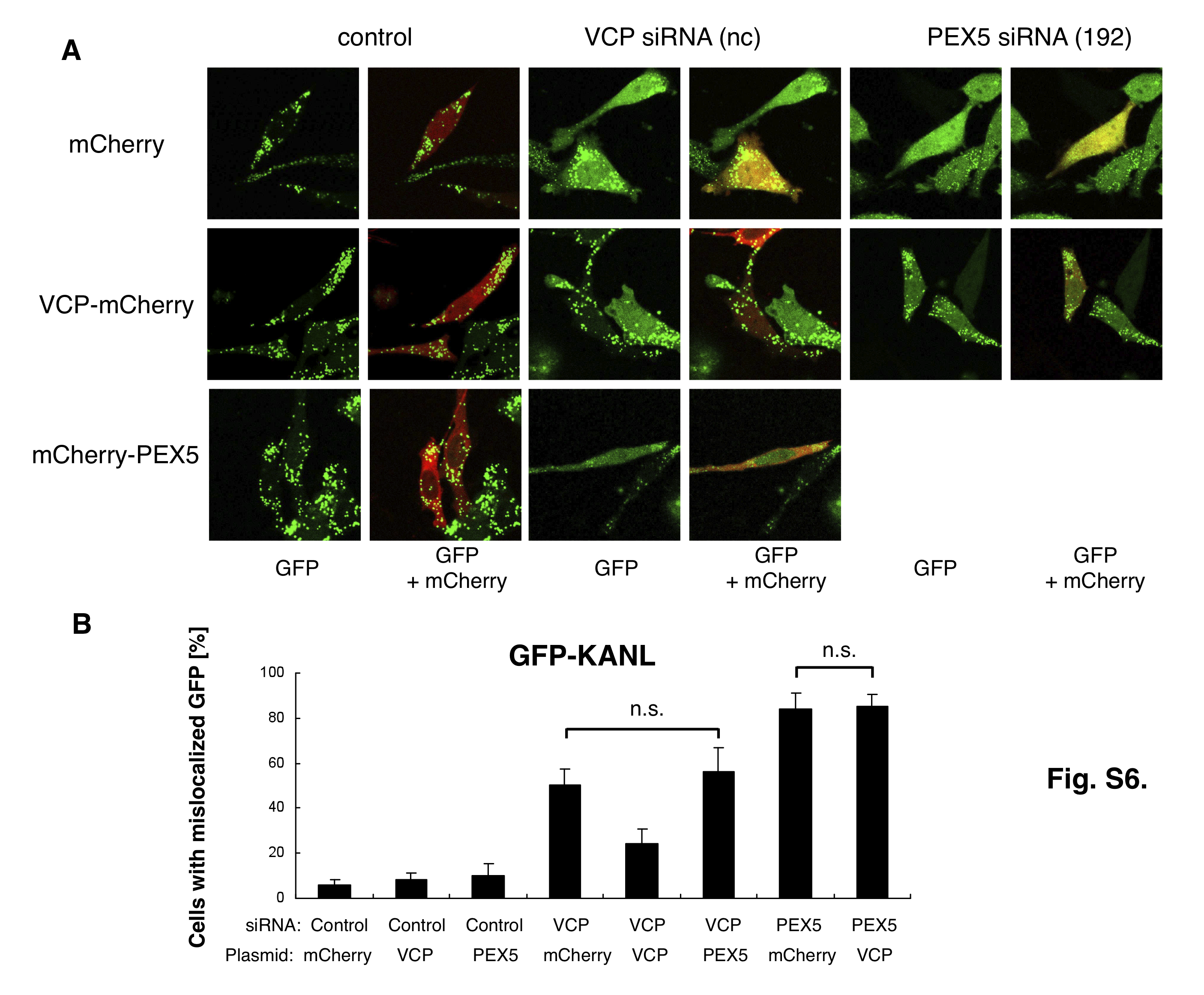

Supplement: Figure S6 — Fluorescence microscopy analysis of intracellular localization of GFP-KANL. (A) HeLa cells continuously expressing GFP-KANL were treated with control siRNA (control), VCP siRNA (nc), or PEX5 siRNA (192) for 48 hours, and then transfected with an expression vector for mCherry, VCP-mCherry, or mCherry-PEX5. Twenty-four hours later, GFP signals (green) and mCherry signals (red) were examined. (B) Quantification of fluorescence microscopy of GFP-KANL in (A). More than 120 mCherry-positive cells were examined in each sample, and the fraction (%) of cells with diffuse GFP signals in the cytoplasm were scored. n.s., not significant. (TIFF) [file pone.0056012.s006.tiff]
